# Análisis de genotipos, alelos y haplotipos de HLA en un grupo de pacientes con artritis psoriásica: poca frecuencia de alelos comunes

**DOI:** 10.7705/biomedica.7555

**Published:** 2024-12-23

**Authors:** Omar-Javier Calixto, María-Alejandra Meneses-Toro, Paula Andrea Chacón, Mónica Acevedo-Godoy, Luisa Constanza Robayo, Juan Manuel Bello-Gualtero, Wilson Bautista-Molano, Verónica Noguera, Jaime Cortés, Consuelo Romero-Sánchez

**Affiliations:** 1 Servicio de Reumatología e Inmunología, Hospital Militar Central, Bogotá, D. C., Colombia Hospital Militar Central Servicio de Reumatología e Inmunología Hospital Militar Central Bogotá Colombia; 2 Grupo de Inmunología Clínica Aplicada, Hospital Militar Central, Universidad Militar Nueva Granada, Bogotá, D. C., Colombia Universidad Militar Nueva Granada Grupo de Inmunología Clínica Aplicada Hospital Militar Central Universidad Militar Nueva Granada Bogotá, D. C. Colombia; 3 Grupo de Inmunología Celular y Molecular - InmuBo, Universidad El Bosque, Bogotá, D. C., Colombia Universidad El Bosque Grupo de Inmunología Celular y Molecular - InmuBo Universidad El Bosque Bogotá, D. C. Colombia; 4 Servicio de Dermatología, Hospital Militar Central, Bogotá, D. C., Colombia Hospital Militar Central Servicio de Dermatología Hospital Militar Central Bogotá, D. C. Colombia; 5 Laboratorio Clínico, Hospital Militar Central, Bogotá, D. C., Colombia Hospital Militar Central Laboratorio Clínico Hospital Militar Central Bogotá, D. C. Colombia; 6 Servicio de Dermatología, Fuerza Aérea Colombiana, Bogotá, D. C., Colombia Fuerza Aérea Colombiana Servicio de Dermatología Fuerza Aérea Colombiana Bogotá, D. C. Colombia; 7 Programa de Medicina Interna, Facultad de Medicina, Universidad Militar Nueva Granada, Bogotá, D. C., Colombia Universidad Militar Nueva Granada Programa de Medicina Interna Facultad de Medicina Universidad Militar Nueva Granada Bogotá, D. C. Colombia

**Keywords:** artritis psoriásica, espondiloartritis, antígenos HLA, genotipo, Arthritis, psoriatic, spondylarthritis, HLA antigens, genotype

## Abstract

**Introducción.:**

La artritis psoriásica es una enfermedad compleja y los antígenos leucocitarios humanos (*Human Leukocyte Antigen*, HLA) son un factor clave en el desarrollo de la enfermedad. En Latinoamérica, hay pocos datos sobre dichos antígenos en pacientes con artritis psoriásica.

**Objetivo.:**

Describir la frecuencia de genotipos, alelos y haplotipos de los antígenos leucocitarios humanos en casos de artritis psoriásica y asociarlos con variables clínicas.

**Materiales y métodos.:**

Se desarrolló un estudio retrospectivo del 2012 al 2023 en el que se evaluaron los adultos con artritis psoriásica según los criterios CASPAR. Se incluyeron controles sanos con HLA-A, B, C y DR tipificados por PCR/SSO en un dispositivo Luminex 100/200 xMAP™. Se hizo un análisis comparativo entre los datos de los antígenos leucocitarios de los pacientes y los de los controles sanos.

**Resultados.:**

Se incluyeron 401 controles sanos y 37 pacientes con artritis psoriásica, entre los cuales se identificaron 46 genotipos, 75 alelos y 32 haplotipos. Los HLA más frecuentes fueron HLA-A*24 (37,1 %), HLA-B*35 (20,8 %), HLA-C*03 y HLA-C*07 (cada uno con el 19,9 %) y HLA-DR*04 (30 %). Al comparar su frecuencia con la de los controles sanos, la frecuencia genotípica fue menor para HLA-A*02, HLA-A*11, HLA-B*35, HLA-DR*01, HLA-DR*07, HLA-DR*13 y HLA-DR*15 (p < 0,05), lo que significa que, si bien el HLA-B*35 fue frecuente en la artritis psoriásica, su frecuencia fue menor al compararse con la de los controles.

La frecuencia de HLA-A*24 y HLA-B*44 fue diferente en los pacientes con compromiso cutáneo (p < 0,05); la de HLA-B*40 y HLA-B*35 en aquellos con compromiso articular (p < 0,05), y la de HLA-A*26 y HLA-C*16 en aquellos con manifestaciones extraarticulares (p < 0,05). La frecuencia alélica de HLA-A*26:01 y HLA-C*16:01 en los casos con manifestaciones extraarticulares, fue también significativa. La frecuencia de HLA-Cw*6 fue 6,7 % y la ausencia de HLA-B*27 en nuestra población es uno de los aspectos por destacar.

**Conclusiones.:**

En el análisis de HLA en artritis psoriásica se encontrótró una poca frecuencia de HLA-C*06 y ausencia de HLA-B*27. Este hallazgo es diferencial respecto a la población caucásica. Estos resultados revelaron otros alelos de interés, algunos relacionados con variables clínicas. Las diferencias encontradas podrían estar relacionadas con la mezcla racial de nuestra población.

## Abstract (en)

**Introduction.:**

Psoriatic arthritis is a complex disease, and human leukocyte antigens (HLA) are key to its development. Latin America and, specifically, Colombia, has scarce data about patients with psoriatic arthritis.

**Objectives.:**

To describe the genotypic, allelic and haplotypic frequency of HLA alleles in psoriatic arthritis and associate them with clinical variables.

**Materials and methods.:**

We conducted a retrospective study involving adult patients with psoriatic arthritis, evaluated according to CASPAR criteria, between 2012 and 2023. We included healthy donors whose HLA-A, B, C, and DR were genotyped by PCR/SSO in a Luminex 100/200 xMAP™ device. We performed an HLA comparative analysis between healthy donors and psoriatic arthritis patients.

**Results.:**

We included 401 healthy controls and 37 patients with psoriatic arthritis, in which we identified 46 genotypes, 75 alleles, and 32 haplotypes. The most frequent HLA were HLA-A*24 (37.1%), HLA-B*35 (20.8%), HLA-C*3 and HLA-C*7 (19.9% each), and HLA- DR*4 (30%). Compared to healthy donors, the patient’s genotypic frequency was lower for HLA-A*02, HLA-A*11, HLA-B*35, HLA-DR*01, HLA-DR*07, HLA-DR*13, and HLA-DR*15 (p < 0.05), which means that even though HLA-B*35 was frequent in psoriatic arthritis, it's frequency was lower when compared to that of healthy controls.

The frequency of HLA-A*24 and HLA-B*44 was different in cutaneous involvement (p < 0.05), HLA-B*40 and HLA-B*35 in joint involvement (p < 0.05), and HLA-A*26 and HLA-C*16 in extra-articular manifestations (p < 0.05). The allelic frequency of HLA-A*26:01 and HLA-C*16:01 in extra-articular manifestations was also significant. The frequency of HLA-Cw*6 was 6.7% and the allele HLA-B*27 was absent.

**Conclusions.:**

The HLA analysis in psoriatic arthritis showed a low frequency of HLA-C*06 and absence of HLA-B*27, different from the information reported for Caucasian population. These results also revealed other alleles of interest. Found differences could be related to the important racial mixing of our population.

La artritis psoriásica forma parte del grupo heterogéneo de espondiloartritis y se caracteriza por la desregulación del sistema inmunológico, una fisiopatología compleja y un amplio espectro de manifestaciones clínicas ^1^. La prevalencia de la artritis psoriásica a nivel mundial varía entre el 0,3 y el 1 % ^2^. Aproximadamente, el 30 % de los pacientes con psoriasis desarrollan artritis psoriásica ^3^, y la tasa de aparición varía entre el 7 y el 27 % ^4^. Sin embargo, se estima un subdiagnóstico hasta del 50 % de los casos ^5^, razón por la cual es crucial reconocer los cambios inflamatorios articulares en casos de psoriasis ^6^.

La artritis psoriásica es el resultado de una compleja interacción entre factores genéticos, ambientales e inmunitarios. En los tejidos afectados por la enfermedad, la importancia crítica de las moléculas del antígeno leucocitario humano (*Human Leukocyte Antigen*, HLA) de clase I (HLA-A, HLA-B y HLA-C) y de su presentación antigénica a los linfocitos T CD8^+^, se ha evidenciado con el aumento de este tipo de célula ^7^. Otros modelos propuestos sugieren que las células presentadoras de antígenos interactúan con células linfoides innatas y linfocitos T vírgenes con función sobre los linfocitos Th1 y Th17. Esta red de interacciones puede determinar los diferentes fenotipos clínicos de la artritis psoriásica ^8^. Por esta razón, los estudios del genoma completo, la heredabilidad y la frecuencia alélica, se han enfocado en estudiar el componente genético de los pacientes con artritis psoriásica que, a su vez, puede diferir de aquel de los pacientes con psoriasis. Por ejemplo, en gemelos y familiares de primer grado, la frecuencia de artritis psoriásica es hasta del 55 %, en contraste con la de psoriasis que es del 10% ^3^.

Se ha encontrado que los alelos que se relacionan con un mayor riesgo de desarrollar artritis psoriásica son HLA-B*08, HLA-B*27, HLA-B*38 y HLA-B*39 ^9^. Casi el 25 % de los pacientes con artritis psoriásica tienen el alelo HLA-B*27 y hasta el 45,9 % de aquellos con psoriasis tienen el HLA-Cw*6 ^10^^,^^11^. La asociación con el HLA-Cw*6 se ha descrito más frecuentemente en la psoriasis con compromiso exclusivamente cutáneo ^3^. No obstante, el subtipo HLA-Cw*06:02 es más común en la artritis psoriásica que en la población general, y se asocia con una edad más temprana de aparición de la enfermedad ^9^. Por otro lado, el alelo HLA-B*27 se ha correlacionado con un menor intervalo de tiempo entre la aparición de las manifestaciones dérmicas y la aparición de las musculoesqueléticas ^1^^,^^12^^,^^13^.

Algunos alelos del HLA están asociados con diferentes manifestaciones clínicas de la artritis psoriásica, por ejemplo: sacroilitis simétrica (HLA-B*27:05), sacroilitis asimétrica (HLA-B*08:01 y HLA-C*07:01), entesitis (HLA-B*27:05 y HLA-C*01:02), dactilitis (HLA-B*27:05 y HLA-B*08:01) y sinovitis (HLA-B*08:01) ^9^.

En Latinoamérica, existe poca información sobre la frecuencia de los genotipos, de los alelos y haplotipos del complejo mayor de histocompatibilidad relacionados con el desarrollo de artritis psoriásica, por lo cual es importante identificar su frecuencia y su asociación con las manifestaciones clínicas.

El objetivo de este estudio fue describir la frecuencia de genotipos, alelos y haplotipos del antígeno leucocitario o HLA en pacientes con diagnóstico de artritis psoriásica y compararlos con los de controles sanos; y además, establecer su asociación con las variables clínicas.

## Materiales y métodos

### Población

Se incluyeron pacientes mayores de 18 años con artritis psoriásica que cumplían los criterios de clasificación CASPAR ^14^. A estos pacientes se les atendió entre el 2012 y el 2023 en el servicio de reumatologia de una institución de referencia en Bogotá (Colombia), y se les tipificó el HLA. Se excluyeron aquellos con otra enfermedad autoinmunitaria concomitante, o con espondiloartritis y sin psoriasis. Los controles sanos fueron personas voluntarias, no emparentadas con los casos, sin infecciones, neoplasias o síntomas articulares, ni signos de enfermedades autoinmunitarias reumáticas o dermatológicas, y con características sociodemográficas similares a las de los pacientes.

Para la selección de los participantes, se utilizó un muestreo no probabilístico por conveniencia. Cuatro investigadores revisaron las historias clínicas durante la fase de preselección. Posteriormente, un reumatólogo y un dermatólogo confirmaron la inclusión de los sujetos. Asimismo, todos los resultados de genotipificación del HLA (HLA-A, HLA-B, HLA-C y HLA-DR) fueron revisados y validados por dos bacteriólogas expertas.

El proyecto fue aprobado por el Comité de Investigación y el Comité de Ética institucional (Código No. 2022-014 y 2017-023).

### Tipificación de HLA

Se extrajeron muestras de sangre y la genotipificación de HLA se hizo mediante la técnica de reacción en cadena de la polimerasa en la que se amplifica el ADN y luego se híbrida con oligonucleótidos específicos de secuencia (PCR/SSO) de los kits LIFECODES™. Las sondas reconocen el polimorfismo del segundo y tercer exón de los genes HLA clase I y II, y los resultados se analizan en el sistema Luminex 100/200 xMAP™ ^15^.

### Análisis estadístico

Las frecuencias de las variables demográficas, genéticas y clínicas se obtuvieron mediante conteo directo.

Se analizaron las variables cuantitativas por medidas de tendencia central y de dispersión. Las variables cualitativas se representaron mediante frecuencias y porcentajes. Se hicieron pruebas de bondad de ajuste normal y diagramas de distribución para cada una de las variables. Las variables cuantitativas con distribución no normal se reportaron con medianas y cuartiles.

Se hizo un análisis bivariado aplicando la prueba de ji al cuadrado o la prueba exacta de Fisher para las variables cualitativas y la prueba de U de Mann-Whitney para las variables cuantitativas no paramétricas. Todos los análisis se realizaron con el *software* estadístico R Studio, versión 2024.04.2.

## Resultados

Se incluyeron 401 controles sanos con una media de edad de 37,8 ± 15,6 años, de los cuales el 48,1 % eran de sexo masculino. Se incluyeron 37 pacientes con diagnóstico de artritis psoriásica con una media de edad de 45,9 ± 16,3 años; 54,1 % eran del sexo masculino. La edad de diagnóstico de la artritis psoriásica tuvo una mediana de 31 años (rango: 21 a 42,3).

Todos los pacientes con artritis psoriásica tuvieron compromiso cutáneo; el 48,7 % tuvo compromiso articular axial, el 75,7 %, articular periférico, el 56,8 %, ungular, y el 9 %, extraarticular.

Respecto a la frecuencia y el tipo de tratamiento, el 86 % de estos pacientes recibió manejo tópico, el 89 % tratamiento sistémico y el 44 % antiinflamatorios no esteroideos. Los pacientes recibieron fármacos antirreumáticos modificadores de la enfermedad sintéticos (subclase uno) o dirigidos a blancos específicos, como metotrexato (83,3 %), sulfasalazina (17,1 %), leflunomida (20 %), tofacitinib y upadacitinib (cada uno 2,9 %). El porcentaje de fármacos antirreumáticos modificadores de la enfermedad biológicos administrados fue del 47,2 %, y los inhibidores del factor de necrosis tumoral alfa (TNF-α) fueron los más frecuentes, seguidos de los inhibidores de la interleucina 17 (IL-17). Las demás características de la población se encuentran en los cuadros 1 y 2.

Se evaluaron las frecuencias alélicas de HLA de 37 pacientes con artritis psoriásica (considerando HLA-A, HLA-B, HLA-C y HLA-DR) y 401 controles sanos (tipificando solamente HLA-A, HLA-B y HLA-DR). La distribución de los alelos de los controles sanos se observa en la figura suplementaria 1.

Se identificaron 75 alelos en los pacientes con artritis psoriásica: 14 alelos de HLA-A, y los más frecuentes fueron HLA-A*24:02 (10,8 %) y HLA-A*31:01 y HLA-A*32:01 (4,1 % cada uno); 29 alelos de HLA-B, y los más frecuentes fueron HLA-B*35:43 y HLA-B*40:02 (6,8 % cada uno), seguidos por HLA-B*14:02 (5,4 %). No se encontraron individuos con HLA-B*27. Se detectaron 13 alelos de HLA-C, y los más frecuentes fueron HLA-C*07:01 y HLA-C*12:03 (2,7 % cada uno); y, finalmente, se identificaron 19 alelos de HLA-DR, y los más frecuentes fueron HLA-DR*04:04 y HLA-DR*04:07 (5,4 % cada uno) (cuadro suplementario 1).

El análisis dio como resultado 32 haplotipos que incluyeron los alelos de HLA-A, B, C y DR, sin encontrar frecuencias superiores al 1 %. Dado el interés en los alelos HLA-B asociados con la enfermedad psoriásica, el análisis de estos mostró un total de 27 haplotipos, todos únicos. Se destacan las agrupaciones con HLA-B*35: HLA-B*35:44-HLA-B*51:10, HLA-B*35:40- HLA-B*40:02, HLA-B*35:44-HLA-B*51:10, HLA-B*35:01-HLA-B*40:01, HLA-B*35:12-HLA-B*45:01, HLA-B*35:43-HLA-B*51:10, HLA-B*35:45- HLA-B*56:01 y HLA-B*35:04-HLA-B*57:03; y aquellas con HLA-B*14: HLA-B*14:02-HLA-B*14:54, HLA-B*14:02-HLA-B*51:01, HLA-B*14:02- HLA-B*44:02 y HLA-B*14:02- HLA-B*35:43 (cuadro suplementario 2).

### Análisis de alelos de HLA en pacientes con artritis psoriásica comparados con los controles sanos

**Figura 1.**
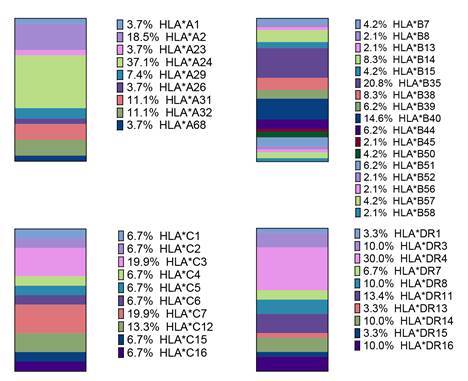
Frecuencias genotipicas del FILA de pacientes con artritis psoriásica (N = 37)

Los genotipos de HLA más frecuentes en el grupo de artritis psoriásica para el alelo HLA-A fueron A*24 (37,1 %), A*2 (18,5 %), A*31 y A*32 (11,0 %); para el alelo HLA-B fueron B*35 (20,8 %), B*40 (14,6 %) y B*14 (8,3 %); para los alelos HLA-C fueron el C*3 y C*7 (19,9 %) y el C*12 (13,3 %); y para HLA-DR los más representativos fueron DR*4 (30,0 %), D*11 (13,4 %) y DR*14, DR*16 y DR*3 (10,0 %) (figura 1).

Al comparar los antígenos entre el grupo de HLA-A, se identificó una diferencia estadísticamente significativa para HLA-A*2 (artritis psoriásica = 6,8 %; controles sanos = 17,8 %; p = 0,004), y HLA-A*11 (artritis psoriásica = 0 %; controles sanos = 4,1 %; p = 0,048).

**Cuadro 1:** Características demográficas y clínicas de los pacientes con diagnóstico de artritis psoriásica (N = 37)

Variables		Valor
Edad (años)		45,8 ± 16,3
Sexo (masculino)		20
Edad al diagnóstico (años)		31 (21 - 42,3)
Comorbilidades		28
Antecedente familiar de psoriasis		3
Antecedente familiar de espondiloartritis		1
Compromiso articular		
	Axial	18
	Periférico	28
	Dolor lumbar inflamatorio	19
	Rigidez matinal	15
	Dolor lumbar mecánico	18
	Dactilitis	6
	Entesitis	14
	Artritis	24
	Artralgias	29
Compromiso cutáneo		
	Psoriasis vulgar/placas	31
	Psoriasis guttata	2
	Psoriasis inversa	3
	Psoriasis ungular	17
Tipo de compromiso ungular		
	Pitting/hoyuelos	9
	Leuconiquia	3
	Puntos rojos en la lúnula	2
	Resquebrajamiento de la lámina o traquioniquia	2
	Hemorragia en astilla	5
	Mancha en aceite	11
	Hiperqueratosis subungular	3
	Onicólisis	12
	No menciona compromiso de uñas	16
Localización del compromiso cutáneo		
	Cuero cabelludo	16
	Cara	6
	Cuello	4
	Miembros inferiores (excepto ingles)	22
	Miembros superiores (excepto axilas)	23
	Tronco	18
	Ombligo	2
	Genitales	4
	Axilas	2
	Ingles	4
	Inframamario	1
	Interglúteo	5
	Palmar	2
	Plantar	1
Compromiso extraarticular		
	Uveítis	1
	Enfermedad de Crohn	1
	Úlceras orales	2

**Cuadro 2:** Características clinimétricas y tratamiento de los pacientes con diagnóstico de artritis psoriásica (N = 37)

Clinimetría		
	*Bath Ankylosing Spondylitis Functional Index -* BAS FI	5,9 ± 0,7
	*Bath Ankylosing Spondylitis Disease Activity Index -* BASDAI	5,8 ± 1,7
	*Axial Spondyloarthritis Disease Activity Score -* AS DAS	2,5 ± 0,5
	*Classification for Psoriatic Arthritis -* CASPAR	3,0 (3-3,5)
	*Disease Activity in Psoriatic Arthritis -* DAPSA	14,8 ± 10,1
	*Nail Psoriasis Severity Index -* NAPS I	4,0 (0,5-11)
	*Dermatology Life Quality Index -* DLQI	7,7 ± 5,6
	*Psoriasis Area Severity Index -* PASI	3,6 (1,6 - 7)
Tratamiento		
Tópico		32
	Corticoide	31
	Tacrolimus	9
	Retinoide	3
	Betametasona y calcipotriol	11
	Betametasona y ácido salicílico	15
Tratamiento sistémico		33
	AINES	16
	Corticoide sistémico	6
	Metotrexate	31
	Sulfasalazina	6
	Leflunomida	7
	Tofacitinib	1
	Upadacitinib	1
Terapia biológica		17
	Secukinumab	7
	Ixekizumab	2
	Golimumab	4
	Adalimumab	10
	Ustekinumab	1
	Infliximab	2
	Etanercept	6
	Guselkumab	1

AINES: antiinflamatorios no esteroideos

**Figura 2.**
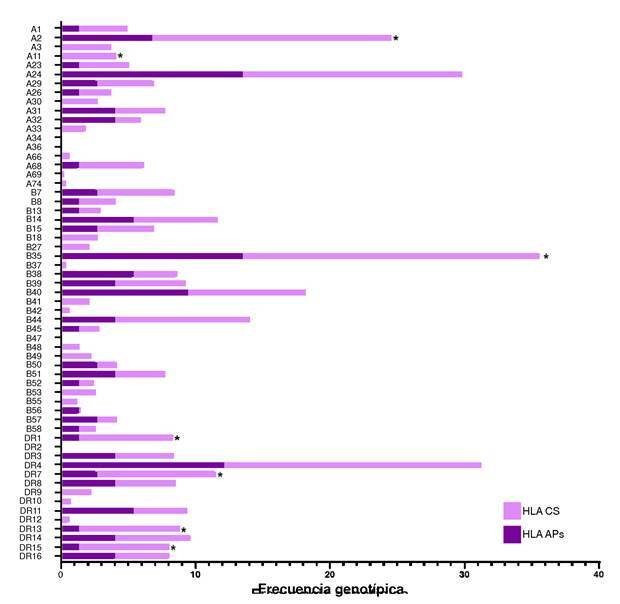
Comparación de frecuencias alélicas de HLA-A, HLA-B y HLA-DR de pacientes con artritis psoriásica (N = 37) y las de los controles sanos (N = 401) CS: controlsano; APs: artritis psoriásica * p valor < 0,05, por la prueba de ji al cuadrado o la prueba exacta de Fisher

 En el análisis de los antígenos de HLA-B, se identificó una diferencia estadísticamente significativa para HLA-B*35 (artritis psoriásica = 13,5 %; controles sanos = 22,1 %; p = 0,031); y para los alelos de HLA-DR, se identificó una diferencia estadísticamente significativa para HLA-DR*1 (artritis psoriásica = 1,4 %; controles sanos = 7,0 %; p = 0,032), HLA-DR*7 (artritis psoriásica = 2,7 %; controles sanos = 8,9 %; p = 0,035), HLA-DR*13 (artritis psoriásica = 1,4 %; controles sanos = 7,5 %; p = 0,023) y HLA-DR*15 (artritis psoriásica = 1,4 %; controles sanos = 6,7 %; p = 0,038) (figura 2). 

Para el caso del HLA-B*27, se obtuvo una frecuencia en artritis psoriásica del 0 % comparada con la de los controles sanos que fue del 2,2 % y resultó sin significancia estadística.

### Análisis de la población con artritis psoriásica según las manifestaciones clínicas

En los pacientes con artritis psoriásica y compromiso cutáneo, se observó que aquellos que presentaban afección de los miembros inferiores, excluyendo la región inguinal, tenían mayor frecuencia alélica de HLA-A*24 (p = 0,023). Se identificó mayor frecuencia de HLA-B*44 en los pacientes con compromiso interglúteo (p = 0,045), aunque estuvo ausente en aquellos con compromiso de miembros superiores, excluyendo la región axilar (p = 0,047).

En aquellos con compromiso articular, se identificaron diferencias estadísticamente significativas en la frecuencia alélica de HLA-BM0 en pacientes con artritis periférica (p = 0,029), mientras que la frecuencia de HLA-B*35 fue significativamente menor en pacientes con artralgias (p = 0,021) o dolor lumbar no inflamatorio (p = 0,038).

Los pacientes con manifestaciones extraarticulares tuvieron una frecuencia alélica significativamente mayor de HLA-A*26 (p = 0,027) y HLA-C*16 (p = 0,016) para uveítis y enfermedad de Crohn (p = 0,027 para los dos alelos). Se encontró una mayor frecuencia de HLA-A*31 y HLA-A*32 en aquellos pacientes con antecedentes familiares de psoriasis (p = 0,015 en los dos alelos). La frecuencia alélica de HLA-Cw*6 se encontró en el 6,7 % de los pacientes, sin asociarse con variables clínicas de interés. No se identificaron pacientes con artritis psoriásica y el alelo HLA-B*27.

La variante alélica HLA-C*07:56 fue más frecuente en los pacientes con compromiso cutáneo inframamario (p = 0,027), mientras que la frecuencia de HLA-DR*03:02 fue significativamente menor en los pacientes con artralgias (p = 0,042). En los pacientes con manifestaciones extraarticulares, las variantes alélicas más frecuentes fueron HLA-A*16:01 (p = 0,027) y HLA-A*26:01 (p = 0,027) para uveítis y enfermedad de Crohn; y HLA-A*31:02 (p = 0,015) y HLA-A*32:01 (p = 0,015) en aquellos con antecedente familiar de psoriasis.

## Discusión

El HLA de clase I es uno de los factores principales descritos de riesgo genético para el desarrollo de artritis psoriásica. En un metaanálisis de estudios genómicos se han identificado más de 20 variantes significativas, incluyendo los alelos de HLA-A, HLA-B, HLA-C y los genes de las interleucinas IL-12B, IL-23R y IL-23A, lo que refuerza el papel clave del componente genético en la artritis psoriásica ^16^^,^^17^.

Los resultados del presente estudio evidenciaron diferencias significativas para los alelos HLA-A*2, HLA-A*11, HLA-B*35, HLA-DR*1, HLA-DR*7, HLA- DR*13 y HLA-DR*15. Al comparar estos hallazgos con los de poblaciones de Canadá, se encontró que las frecuencias alélicas eran diferentes, con predominio de HLA-A*1 y HLA-A*26, sin diferencias significativas con los controles sanos ^18^. Sin embargo, de manera similar a los resultados del grupo de pacientes con artritis psoriásica, en un estudio del Reino Unido se encontró una mayor frecuencia para HLA-A*2 y no para HLA-A*11 ^19^. Esto también concuerda con un estudio colaborativo internacional que incluyó 1.962 pacientes con artritis psoriásica y 8.923 controles, en el cual se reportó una diferencia estadísticamente significativa para la variante alélica HLA-A*02:01 ^17^, encontrada con mayor frecuencia en los pacientes evaluados.

En cuanto a la frecuencia alélica del grupo HLA-B, en diversos estudios se han encontrado asociaciones con alelos que confieren un riesgo específico para la artritis psoriásica, como HLA-B*08, HLA-B*27 y HLA-B*38 ^20^. Sin embargo, ninguno de estos alelos mostró significancia estadística en el grupo evaluado de pacientes con artritis psoriásica. Solo se encontró una diferencia estadísticamente significativa para HLA-B*35, mientras que HLA-B*40 y HLA-B*13, si bien fueron frecuentes, no alcanzaron diferencias estadísticas, lo que concuerda con datos previos de poblaciones de Colombia ^21^, Uruguay ^22^, Argentina ^23^, y lo reportado en la población de origen hispano de los Estados Unidos ^24^.

La frecuencia del HLA-B*27 ha sido ampliamente estudiada en la espondiloartritis, pero en la artritis psoriásica sigue siendo motivo de controversia. En estudios anteriores, se ha reportado una frecuencia del 35 % en pacientes de Estados Unidos ^25^, 39 % en Taiwán ^26^, 17 % en España ^27^ y 4 % en Israel ^28^. En Latinoamérica, particularmente en Brasil, la frecuencia reportada es del 27,3 % ^29^, mientras que en otra serie de pacientes colombianos se identificó una frecuencia del 23 % ^30^.

En este trabajo, HLA-B*27 estuvo ausente en los 37 pacientes evaluados, lo que coincide con un estudio previo en Colombia, en el cual tampoco se encontró este alelo en los pacientes con espondiloartritis ^31^. Se destaca el predomino del compromiso articular periférico sobre la axial, estando presente en el 76 % de nuestra población, lo cual es compatible con lo reportado en la región con bajas frecuencias de HLA-B*27 ^31^^-^^33^.

La relación de alelos de HLA con manifestaciones cutáneas proviene del análisis de los pacientes con psoriasis y no con artritis psoriásica, y varía según la población estudiada. En los pacientes de Asia, predominan HLA-A*30, Cw*06 y DR*07 ^34^. En los pacientes del Medio Oriente, se describió que la variante HLA-DRB1*11:01 se ha asociado significativamente con la psoriasis en placas y, la HLA-DRB1 *01:02 con otros tipos de psoriasis, mientras que la HLA-DRB1 *03:06 se ha asociado con todos los tipos de psoriasis ^16^. Además, en India se reportó que HLA-A*1, HLA-A*24, HLA-A*28, HLA-A*30, HLA-B*35, HLA-Cw*6, HLA-DR*3 y HLA-DQ*1 eran más comunes en los pacientes con psoriasis ^35^. Por otro lado, se ha descrito que HLA-DR*3 y HLA-DR*53 están presentes con mayor frecuencia en los pacientes con psoriasis ^36^. No se encontraron reportes de la asociación entre las manifestaciones cutáneas según su localización y los alelos de HLA de los pacientes con artritis psoriásica.

En el presente estudio, la distribución cutánea podría estar influenciada por el HLA-A*24, previamente reportado en India ^35^, y el HLA-B*44. No obstante, es importante destacar la heterogeneidad del compromiso cutáneo que también depende de la gravedad de la enfermedad ^1^^,^^37^. Se requieren estudios en poblaciones más grandes para corroborar estos hallazgos.

La relación entre los alelos de HLA y las manifestaciones articulares en los pacientes con artritis psoriásica es heterogénea. Se reportó una asociación significativa entre la afectación de las articulaciones interfalángicas distales y HLA-A*26 y HLA-B*38, y la afectación inflamatoria axial y el HLA-B*35 ^28^. En la población descrita, la frecuencia de HLA-B*35 fue significativamente menor en pacientes con presencia únicamente de artralgias o dolor lumbar, pero no en aquellos con manifestaciones inflamatorias.

La variante alélica HLA-DR*03:01 se ha asociado con afectación axial, presentándose dolor lumbar inflamatorio en el 84 % de los casos frente al 25 % de los pacientes con HLA-DR*03:01 ^28^. Aunque HLA-DR*01:01 mostró una diferencia estadística significativa al compararlo con el de los controles sanos, no se identificó ninguna característica clínica específica relacionada con este antígeno. La distribución de los alelos de HLA fue similar dentro de los grupos de pacientes con oligoartritis o poliartritis ^28^. Se identificó una mayor frecuencia alélica de HLA-BMO en los pacientes con artritis periférica.

La frecuencia de HLA-A*26 y HLA-C*16 fue significativamente mayor en los pacientes con manifestaciones extraarticulares, como uveítis y enfermedad de Crohn. Aunque en la artritis psoriásica esta asociación no se ha descrito, recientemente se reportó una similar para el compromiso gastrointestinal y la uveítis en la enfermedad de Behçet ^38^, que también hace parte del espectro de las enfermedades autoinflamatorias. En Bélgica, describieron una prevalencia mayor de HLA-B*27, HLA-Bw*62 y HLA-B*17 en los pacientes con artritis psoriásica: HLA-B*27 y HLA-Bw*62 fueron más frecuentes en los pacientes con enfermedad inflamatoria intestinal, el 60 y el 50 %, respectivamente ^39^.

Solo se encontraron dos estudios que evaluaron la uveítis: uno en Japón, en el que seis casos portaban HLA-A*2, cuatro casos con HLA-B*46 y ningún caso con HLA-B*27 en psoriasis ^40^; y otro en España, en pacientes artritis psoriásica asociada con HLA-DR*13 ^41^. No obstante, la poca frecuencia de estas manifestaciones en la población evaluada y en la literatura científica, sugiere que estas asociaciones deben interpretarse con precaución.

Las diferencias en los resultados podrían estar influenciadas por el intenso flujo genético y cultural que ha experimentado Colombia, que se traduce en una gran diversidad étnica y una notable heterogeneidad entre regiones geográficas ^42^. Esta heterogeneidad se mantiene en la población actual, lo que apoya las diferencias encontradas en los alelos de HLA en pacientes con artritis psoriásica comparadas con las de otras poblaciones ^43^.

En este estudio, se reportan las frecuencias alélicas de HLA-A, HLA-B, HLA-C y HLA-DR, y los haplotipos de pacientes colombianos con artritis psoriásica y controles sanos, lo cual contribuye a dilucidar las bases genéticas y los fenotipos clínicos de esta enfermedad. La baja frecuencia de HLA-C*w6 o la ausencia de HLA-B*27 en los pacientes con artritis psoriásica respecto a poblaciones caucásicas, podría estar relacionada con el mestizaje de la población colombiana y con el predominio periférico del compromiso articular, lo que impacta la heterogeneidad de la presentación clínica. Además, es importante destacar que en este estudio se ha logrado identificar alelos de HLA en una población con artritis psoriásica, excluyendo otras espondiloartritis, lo que permite una mayor diferenciación de este fenotipo en particular.

El presente estudio tiene algunas limitaciones, como el número relativamente pequeño de individuos, a pesar de los más de 10 años de evaluación de la artritis psoriásica en la institución de referencia. Aun así, se encontraron diferencias relevantes para la población colombiana. En futuros estudios, se recomienda incluir un tamaño de muestra mayor que permita evaluar de manera más confiable la relación entre las variables.

## Figures and Tables

**Figura suplementaria 1.**
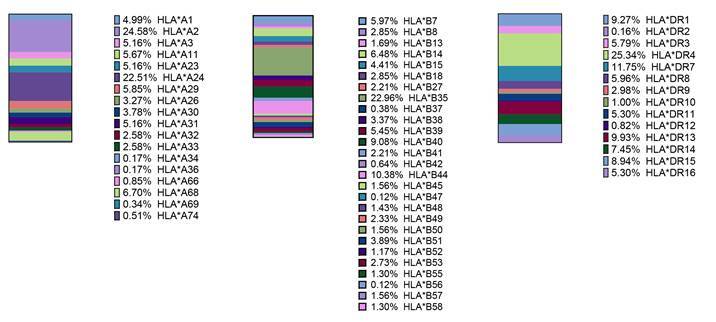
Frecuencias alélicas de HLA-A, HLA-B y HLA-DR de los controles sanos (N = 401)

## Archivos suplementarios

**Cuadro suplementario 1.** Frecuencias alélicas de HLA-A, HLA-B, HLA-C y HLA-DR de pacientes con artritis psoriásica (N = 37)

HLA-A	n	HLA-B	n	HLA-C	n	HLA-DR	n
01:01	1	07:02	1	01:02	1	01:02	1
02:01	3	08:01	1	02:02	1	03:01	1
02:22	1	08:12	1	03:02	1	03:02	1
23:01	1	13:02	1	03:04	1	04:04	2
24:02	4	14:02	2	03:05	1	04:05	1
24:03	1	14:54	1	04:01	1	04:08	1
24:24	1	15:03	1	05:01	1	04:07	2
24:14	1	15:16	1	06:02	1	07:01	1
24:27	1	35:01	1	07:01	1	08:02	1
29:02	1	35:04	1	07:56	1	08:04	1
26:01	1	35:12	1	12:03	1	11:01	1
31:01	2	35:40	1	15:02	1	11:03	1
32:01	2	35:43	3	16:01	1	11:04	1
68:01	1	35:44	1			13:03	1
		38:01	2			14:01	1
		39:01	1			14:02	1
		39:05	1			15:01	1
		40:01	1			16:01	1
		40:02	3			16:02	1
		44:02	1				
		44:03	1				
		45:01	1				
		50:01	1				
		51:01	1				
		51:10	1				
		52:01	1				
		56:01	1				
		57:01	1				
		58:01	1				

**Cuadro suplementario 2.** Haplotipos de HLA identificados de pacientes con artritis psoriásica (N = 37)

Haplotipo 1 (A / B / DR)	Haplotipo 2 (A / B / DR)
26:01 / 38:01 / 07:01	29:02 / 44:03 / 11:03
23:01 / 35:12 / 01:01	24:02 / 45:01 / 04:07
24:02 / 39:01 / 04:07	24:27 / 74:00 / 20:80*2
31:01 / 40:01 / 04:04	32:01 / 44:02 / 11:04
02:01 / 39:01 / 0404	02:22 / 40:02 / 16:02
01:01 / 38:01 / 11:01	02:01 / 39:05 / 16:01
24:02 / 35:43 / 04:07	24:02 / 51:10 / 16:02
24:02 / 35:43 / 04:04	31:01 / 56:01 / 04:07
29:02 / 08:01 / 03:01	32:01 / 08:12 / 31:30*3
24:24 / 35:40 / 04:08	24:02 / 40:02 / 08:02
31:01 / 14:02 / 01:02	32:01 / 35:43 / 04:05
24:02 / 35:44 / 03:02	68:01 / 51:10 / 04:07
24:02 / 40:02 / 04:04	24:27 / 75:80 / 11:40*1
02:01 / 35:04 / 08:04	24:14 / 40:02 / 15:01
02:01 / 38:01 / 07:01	24:03 / 57:03 / 11:03
02:01 / 07:02 / 14:02	24:02 / 50:01 / 14:01
